# Draft Genome Sequences of Four Thermophilic Spore Formers Isolated from a Dairy-Processing Environment

**DOI:** 10.1128/genomeA.00757-16

**Published:** 2016-08-11

**Authors:** Martien P. M. Caspers, Jos Boekhorst, Anne de Jong, Remco Kort, Masja Nierop Groot, Tjakko Abee

**Affiliations:** a Top Institute (TI) Food and Nutrition, Wageningen, The Netherlands; b TNO, Microbiology and Systems Biology, Zeist, The Netherlands; c NIZO food research, Ede, The Netherlands; d Laboratory of Food Microbiology, Wageningen University and Research Centre, Wageningen, The Netherlands; e Wageningen UR Food & Biobased Research, Wageningen, The Netherlands; f Molecular Cell Physiology, Vrije Universiteit (VU) Amsterdam, Amsterdam, The Netherlands; g Molecular Genetics, Rijks Universiteit Groningen (RUG), Groningen, The Netherlands

## Abstract

Spores of thermophilic spore-forming bacteria are a common cause of contamination in dairy products. Here, we report draft genome sequences of four thermophilic strains from a milk-processing plant or standard milk, namely, a *Geobacillus* *thermoglucosidans* isolate (TNO-09.023), *Geobacillus stearothermophilus* TNO-09.027, and two *Anoxybacillus flavithermus* isolates (TNO-09.014 and TNO-09.016).

## GENOME ANNOUNCEMENT

One of the regular problems in the production of dairy concentrates is contamination by heat-resistant spores from thermophilic bacteria of the genera *Anoxybacillus* and *Geobacillus* (1). Genome sequences of two thermophilic strains previously isolated from fouling samples from two dairy-production plants (1) and with relatively strong biofilm-forming capacities, namely, *Geobacillus thermoglucosidans* TNO-09.020 and *Anoxybacillus flavithermus* TNO-09.006, were reported previously (2, 3). Here, we publish the draft genome sequences of an additional set of four dairy thermophiles from a published study (1).

*G. thermoglucosidans* TNO-09.023, *G. stearothermophilus* TNO-09.027, *A. flavithermus* TNO-09.014, and *A. flavithermus* TNO-09.016 were pregrown on tryptic soy agar (TSA) plates (at 55°C overnight, in plastic bags with wet tissues to prevent evaporation). Genomic DNA was isolated from pelleted cell material of freshly grown flask cultures in tryptic soy broth (TSB) (at 55°C, with optical density at 600 nm [OD_600_] of 0.8 to 1.0), inoculated from a single representative freshly grown colony from a TSA plate. The method involved a mild degradation step of cell walls by lysozyme at 37°C, followed by lysis of cells by the addition of sarcosyl. Proteins were removed by extraction with phenol-chloroform, RNA was degraded using RNase, and the resulting DNA was precipitated and washed with isopropanol (50% [vol/vol]) and 70% (vol/vol) ethanol, respectively (4). DNA was dissolved in water (1 to 9 µg/µl; size on 0.8% agarose gel, ≥20 kb) and used for sequencing.

The isolated DNA was sheared to 250- to 350-bp fragments and paired-end sequenced on an Illumina HiSeq 2000 outsourced to BaseClear (Leiden, The Netherlands). Ray 2.3.1 (5) was used for assembly. The RAST server (6) was used to annotate the genomes.

### Accession number(s).

The genome sequences of the four strains have been deposited at DDBJ/EMBL/GenBank under the accession numbers listed in Table 1. The version described in this paper is the first version.

**TABLE 1:** Sequenced strains and their sources

Species	Strain	Source of isolation	Accession no.
*Geobacillus thermoglucosidans*	TNO-09.023	Casein pipeline	LUCT00000000
*Geobacillus stearothermophilus*	TNO-09.027	Casein pipeline	LUCR00000000
*Anoxybacillus flavithermus*	TNO-09.014	Standard milk	LUFB00000000
*Anoxybacillus flavithermus*	TNO-09.016	Evaporator	LUCQ00000000
